# A Plea for the Conservation of the Inferior Turbinate

**Published:** 1914-09

**Authors:** Albert B. Mason

**Affiliations:** Waycross, Ga.


### A PLEA FOR THE CONSERVATION OF THE INFERIOR TURBINATE.

By Albert B. Mason, M. D., Waycross, Ga.

I have nothing new to offer for your consideration. All I shall state is of common knowledge. I simply wish to enter my protest against the too frequent removal of all or part of the inferior turbinate, which I believe is the most important organ in the nose. Yet, it is slaughtered and removed with indiscriminate abandon more than any other part of the body, with the possible exception of the prepuce.

I do not mean to infer that it should never be treated surgically, but I do protest against its removal except when necessary for sinus operations, tear duct disease, or neoplasms, and when it is cystic or hypertrophied. And when I speak of hypertrophy of the turbinate bones, I mean hypertrophy of the mucous membrane covering them.

To arrive at a proper understanding of the question before us, it will be necessary to briefly review the anatomy of the mucous membrane of the nose and the physiology of the nose itself.

The mucous membrane of the nose is continuous with that of the eye, through the lacrymal duct; with that of the maxillary and sphenoidal sinuses, through their several ostia in the meati; and with that of the internal ear, through the Eustachian tube. Below and behind, it is continuous with the mucous membrane of the pharynx.

At the vestibule the mucous membrane is of the pavement variety. It is gradually replaced from before backward by columnar ciliated epithelium which covers the whole of the nose except the olfactory area, a small area situated in the upper part of the nose on the superior turbinate, and a corresponding part of the septum. Here we find mucous membrane of special sense, columnar, but non-ciliated.

Serum secreting glands are found in all parts, but more especially on the lower surface of the middle turbinate and the inner surface of the inferior turbinate. In this location they are very abundant. The vascular supply in this same location is very rich, so much so that a certain degree of engorgement can be demonstrated in every nose. Over the septum the mucous membrane is somewhat thinner and greatly less vascular and glandular.

The three chief functions of the nose are: (1) warming, moistening and cleansing inspired air; (2) acting as a sounding chamber; (3) olfaction. I have mentioned them in what I consider their order of importance.

The main function is to warm, moisten and cleanse inspired air. To this end, the turbinates are arranged so that their large mucous surfaces are in contact with the inspiratory stream. The principal stream of air is directed upward toward the anterior end of the middle turbinate. From here it is thrown downward in a whirling motion against the inner surface of the inferior turbinate and passed on to the posterior nares. Other smaller currents whirl under the inferior turbinate along the floor of the nose; still others pass up into the olfactory area.

The inner, or upper, surface of the inferior turbinate and the lower border of the middle are in contact with the main stream of inspired air, and are chiefly concerned in warming and moistening it, as it is here that the cavernous venous plexus —the so-called erectile tissue—is found, whose abundant supply of glands moistens, and whose rich and active blood supply warms (or cools, as the case may be) the air, before passing it back to the rest of the respiratory tract.

It is by the aid of the ciliated epithelium that particles of dust and other impurities are removed and caused to adhere to the moist surface of the mucous membrane.

As a sounding chamber the nose gives tone and resonance to the voice. It is necessary for the proper performance of this function that the nose be free and open to admit the voice. It is quite common for one to hear the remark that a certain person "talks through his nose," when, in fact, it is because the voice is not thrown through the nose that the person talks with a twang.

Olfaction is dependent, among other things, upon free nasal passages, in order that the volatile substances mixed with the inspired air may come in contact with the olfactory nerve endings.

From this brief review of the physiology of the nose, we find that the three functions of importance are dependent upon free admittance of inspired air.

All cases of intranasal obstruction and impaired drainage, unless due to temporary or acute congestion or constitutional diseases, should be treated surgically. That many of them are treated for months and even years with sprays is sadly true. It is possible, I admit, to get some relief by this method, but the treatment only removes the effect and leaves the cause. And when treatment is stopped, the nose returns to its former condition and the patient is still troubled with difficult breathing, mucous still accumulates, and hawking and spitting are still indulged in.

In practically every case of this kind the first thing noticed upon examination is a swollen inferior turbinate. And many doctors without looking farther are satisfied that they have located the seat of trouble, and a partial or complete removal is the result.

A large swollen turbinate is not necessarily the cause of the obstruction. My experience is that it is very seldom the cause. If not the cause, why is it so large and red? Will not its removal unstop the nose and give the patient relief?

Most frequently this apparent hypertrophy is due to a hypertrophied, cystic or polypoid middle turbinate, a thick septum, or spurs and ridges on the same. Less frequently it is due to the presence of adenoids. Constitutional diseases, such as rheumatism, gout and toxemias, sometimes cause a swelling of the inferior turbinate.

If we remember the direction taken by the main stream of inspired air, which is upward toward the middle turbinate and through the middle fossa to the posterior nares, we will understand why deviations and other abnormalities of the septum and the diseased middle turbinate should receive our attention surgically, and the inferior turbinate left to take care of itself.

Of course, removing part or all of the interior turbinate will give relief, but the larger air space is obtained at the sacrifice of a large area of functionating tissue that is needed to prepare the air for inhalation, and after a short while the patient will suffer from dryness of the nose and throat, accompanied by accumulations of dried mucous, and possibly a hacking cough, all of which is due to the fact that the inspired air is not properly prepared for inhalation.

It is an easy matter to differentiate between a real and a compensatory, or apparent, hypertrophy. A real hypertrophy, if pressure be made upon it with a probe, will retain the indentation for quite a while. A compensatory hypertrophy will immediately resume its shape when the pressure is removed. Spraying the nose with a weak solution of cocain is another, and better method of differential diagnosis. The cocain will cause a shrinkage of all but true hypertrophied mucous membrane.

If I find the large swollen inferior turbinate greatly reduced in size after spraying with the cocain solution, it is all the proof needed to convince me that I have a compensatory hypertrophy to deal with; and a thorough examniation results in finding the real cause of the obstruction to be a thick septum, deviations, spurs or ridges on it, or a hypertrophied, cystic or polyphoid middle turbinate.

Since the introduction of the submucous resection of the septum by Killian, Freer and others, there seems to me to be no excuse for the wholesale removal of the inferior turbinate, in part or in toto for the relief of intranasal obstruction. And, I believe that operations upon the turbinate are being done less frequently than before the submucous operation was originated.

Still, there are some rhinologists who advocate operations upon the turbinate, and some who operate upon the turbinate in nearly every case of obstruction. One writer* states: "If

I have a case of nasal obstruction............and I find a moderately large inferior turbinate and a slightly deflected septum I always attack the turbinate rather than touch the septum," giving as his reasons for this statement, "damage to the mucous membrane, the danger of perforation, flat nose, and synechia formations," following resections.

I must insist that I cannot agree with this view. In the first place, "damage to mucous membrane" is certainly much greater in turbinotomy than in submucous resection. In the former a large area is completely removed, while in the latter the linear incision heals with practically no destrcution of mucous membrane. Furthermore, the mucous membrane covering the inferior turbinate is the most important functionating part of the nose, and for this reason should be left alone, when possible.

In the second place, perforations, flat nose and synechia are rare, the last two extremely so. The majority of perforations are due to unskillful surgery, but some of them are unavoidable, and all operators are going to have a small percentage of them. But, even if we get perforations, we have not damaged the nose as much as we would if we removed a large part of the mucous membrane from the inferior turbinate.

I have time and time again seen large inferior turbinates diminish in size after the removal of spurs that were pressing against them; and, I have also seen a condition resembling atrophic rhinitis follow the removal of part of the turbinate. I have had patients, upon whom turbinotomies had been done some time previously, complain of a dryness of the nose and throat, but I have never seen or heard of a case developing a dryness after a submucous resection.

In conclusion, I plead with you, both rhinologist and general practitioner, to respect the function of the inferior turbinate and to save it when possible to do so.

*Griffin: Turbinotomy, Medical Record, Dec. 20, 1913.

224-226 Bunn Building.

